# Prognosis of Midkine and AT1R expression in resectable head and neck squamous cell carcinoma

**DOI:** 10.1186/s12935-023-03060-z

**Published:** 2023-09-24

**Authors:** Tai-Jan Chiu, Chang-Han Chen, Yi-Ju Chen, Yinshen Wee, Ching-Shuen Wang, Sheng‑Dean Luo

**Affiliations:** 1 Department of Hematology‑Oncology, Kaohsiung Chang Gung Memorial Hospital, and Chang Gung University College of Medicine, Kaohsiung, 833 Taiwan; 2 Department of Medical Research, Taichung Veterans General Hospital, Taichung, Taiwan; 3 Kaohsiung Cancer Prevention and Screening Center, Kaohsiung, 833 Taiwan; 4 Department of Pathology, University of Utah, Salt Lake City, UT 84112 USA; 5 School of Dentistry, College of Oral Medicine, Taipei Medical University, Taipei, 110 Taiwan; 6 Department of Otolaryngology, Kaohsiung Chang Gung Memorial Hospital and Chang Gung University College of Medicine, Kaohsiung, 833 Taiwan; 7 Graduate Institute of Clinical Medical Sciences, College of Medicine, Chang Gung University, Taoyuan, 33302 Taiwan; 8 School of Traditional Chinese Medicine, Chang Gung University College of Medicine, Taoyuan, 33302 Taiwan

## Abstract

**Background:**

Research studies have demonstrated that Midkine (MDK) can influence the expression and activity of Renin-angiotensin system (RAS) components. Angiotensin II is involved in tumor growth and angiogenesis in different cancers. We previously observed Angiotensin II receptor blockers (ARBs) improve the survival rates of patients with oral cancers. These findings have prompted us to investigate whether MDK can influence the RAS pathway, mainly through its association with angiotensin II type 1 receptor (AT1R), which contributes to the observed poor prognosis in head and neck squamous cell carcinoma (HNSCC) patients.

**Methods:**

MDK and AT1R expressions were examined in 150 HNSCC patients post-operation by immunohistochemical staining between 1 January 2010 and 31 December 2016. We tested the over-expression and silencing of MDK to evaluate the AT1R expression and functional biological assays in HNSCC cell lines HSC-3 and SAS.

**Results:**

Positive expression of MDK is correlated with positive AT1R expression. MDK predicted poor NSCC patients’ survival. Silencing MDK could suppress AT1R and pAKT expression and reduce the growth, migration, and invasion of HNSCC cells. ARB also inhibits MDK stimulating HNSCC cell proliferation. Overexpression of MDK could upregulate AT1R and pAKT.

**Conclusions:**

MDK is an independent prognostic factor of HNSCC post-operation, and AT1R regulates HNSCC cell growth, invasion, and migration. Positive MDK and AT1R expressions are highly correlated. Mechanistically, the interaction between MDK and AT1R is crucial for MDK-mediated cell viability, and inhibiting AT1R can effectively counteract or abolish these effects. Furthermore, MDK exerts a regulatory role in the expression of AT1R, as well as in the growth and motility of HNSCC cells. These findings highlight the involvement of the interaction between MDK, AT1R, and the pAkt signaling pathways in HNSCC cell viability growth.

**Supplementary Information:**

The online version contains supplementary material available at 10.1186/s12935-023-03060-z.

## Introduction

More than one million new head and neck cancer patients are diagnosed annually, the seventh most common cancer globally [1]. In Taiwan, head and neck is the sixth most common cancer and the fourth cancer-related death in man. The dominant histological type of head and neck cancer is squamous cell carcinoma. Surgery is the most effective treatment for early and local advanced head and neck squamous cell carcinoma (HNSCC). The prognosis of tumor recurrence and overall survival for HNSCC patients post-operation depends on pathological features, including tumor size, lymph node status, AJCC tumor stage, and extra-nodal extension. Besides, patient-related factors were also correlated with the prognosis of HNSCC patients, such as age, sex, smoking, alcohol, betel nuts, and performance status. Despite progress in diagnosis and treatments, the outcome of HNSCC remains unsatisfactory, even in patients who received complete tumor resection. Identifying new treatment targets is important in HNSCC patients with poor prognostic factors.

The renin-angiotensin system (RAS) is essential in blood pressure control and electrolyte balance. Angiotensin I-converting enzyme inhibitors (ACEIs) and angiotensin II type 1 receptor blockers (ARBs) are RAS antagonists to inhibit the effect of angiotensin II. ACEI and ARBs are the most common in the treatment of chronic hypertension and congestive heart failure [2]. Growing evidence demonstrated that RAS promoted cell proliferation and neovascularization by angiotensin II signaling stimulation of vascular endothelial growth factor (VEGF)-mediated angiogenesis in malignancy [3]. The previous study showed that ACEI and ARBs might inhibit tumor development and progression [4] and a promising anti-tumor strategy. Our previous study also found that advanced HNSCC patients who received ARBs for more than 180 days could improve their overall survival after tumor resection [5]. The mechanisms of RAS inhibitors in patients with HNSCC remain unclear. Angiotensin II, a peptide hormone, has biological effectors in RAS. AT1R and AT2R are two types of angiotensin II play different roles in cardiovascular functions. The RAS was observed to activate angiotensin II and upregulate AT1R expression in some cancers. There is only few studies to discuss about AT1R in HNSCC.

Midkine (MDK), a retinoic acid-inducible heparin-binding growth factor, is a useful biomarker to predict HNSCC survival after surgery in our previous study [6]. MDK expression was upregulated in tumor tissue and was associated with lower recurrence-free and overall survival (OS) rates in this study. MDK plays a role in the multiple biological functions of cancer, such as promoting tumor cell proliferation, transformation, and epithelial-to-mesenchymal (EMT) transition [7–9]. One study also showed that MDK could regulate RAS in mice models [10]. The literature review shows the limited relationship between MDK and RAS in HNSCC. The purpose of this study will identify whether angiotensin receptors (AT1R) regulated head and neck cancer cell proliferation and metastases by MDK expression.

## Material and methods

### Patient population

This retrospective study enrolled 150 HNSCC patients who received tumor resection between 1 January 2010 and 31 December 2016 at Kaohsiung Chang Gung Memorial Hospital Medical Center in Taiwan. Patients with synchronous cancers or receiving preoperative chemotherapy, radiotherapy, or concurrent chemoradiotherapy (CCRT) were excluded. The pathological paraffin blocks and medical information of HNSCC patients were from the Kaohsiung Chang Gung Memorial Hospital biobank. The pathological TNM stage was according to the 7th American Joint Committee on Cancer (AJCC) staging system. Overall survival (OS) was counted from surgery to death due to all causes. Disease-free survival (DFS) was computed from the time of surgery to the recurrence or death of any reason without evidence of recurrence. The study was performed under the Declaration of Helsinki and was approved by the Human Research Ethics Committee of Chang Gung Memorial Hospital.

### Immunohistochemical study

A pathologist reviewed the tissue sample from our hospital’s biobank to confirm the histologic type of squamous cell carcinoma. Immunohistochemistry was used to evaluate the levels of MDK and AT1R proteins from 150 HNSCC patients. The protocol of immunohistochemistry for MDK (Abcam Plc, Cambridge, UK) was according to our previous studies [6]. Immunohistochemistry staining for AT1R (A14201, 1:100, ABclonal, USA) was done using an immunoperoxidase technique as in Li et al. study [11]. Staining was performed on slides of formalin-fixed, paraffin-embedded tissue sections with primary antibodies against AT1R. Antibody assay without the primary antibody was used as the negative control. Two pathologists independently evaluated immunohistochemical staining for MDK and AT1R blinded to the clinical information. The scores of the expression of MDK and AT1R followed the previously published methods [12–14]. Pathologists scored MDK in each specimen from 1 to 4 according to the percentage of positive cells: 1 for ≤ 5% of the cells, 2 for 6—35% of the cells, 3 for 36—70% of the cells, and 4 for ≧ 71% of the cells. In addition, we also assigned each specimen another score from 1 to 4 based on staining intensity: 1 for negative staining, 2 for weak staining, 3 for moderate staining, and 4 for intense staining. We calculated the MDK expression by multiplying the percentage and intensity scores. The **strong** MDK protein expression indicated a score of ≥ 4; otherwise, a score of < 4 was weak. The **strong** expression of AT1R was defined as at least staining≧35% of tumor cells, and < 35% was **weak** expression.

### Western blotting

Cells of HNSCC cell lines were collected and lysed with RIPA buffer (Thermo SCIENTIFIC, Rockford, USA), protease inhibitor cocktail set III (MedChemExpress, HY-K0010), and phosphatase inhibitor cocktail (MedChemExpress, HY-K0021) on ice for 30 min. The clear lysate was harvested by centrifugation at 13,000 rpm for 30 min at 4 °C. The total protein concentration was measured, and equal amounts of protein were separated by SDS-PAGE and then transferred onto PVDF membranes. For blocking, membranes were incubated with 1% PBST containing with 5% non-fat milk for 60 min. Primary antibodies to detect MDK (Abcam Plc, Cambridge, UK), AT1R (ABclonal, USA), pAKT (cell signaling#4060), AKT (cell signaling#9272), and β-actin (Sigma #A5441) were added to the membranes and were then incubated overnight at 4 °C. Horseradish peroxidase-conjugated anti-mouse or anti-rabbit IgG secondary antibodies were added to the membranes and left for 1 h at room temperature the next day. X-ray films explored the proteins.

### Cell lines, cell culture, and transfection

Human HNSCC cell lines CAL27 (RRID: CVCL_1107), SAS (RRID: CVCL_1675) and HSC-3 (RRID: CVCL_1228) were obtained from ATCC (American Type Culture Collection) and cultured in DMEM (Life Technologies, Inc., Carlsbad, USA) supplemented with 10% fetal bovine serum (FBS) (Life Technologies, Inc., Carlsbad, USA), 100 U/ml penicillin and 100 μg/ml streptomycin, 1% non-essential amino acid and 1% sodium pyruvate (Life Technologies, Inc., Carlsbad, USA). In a humidified atmosphere, we cultured cells at 37 °C, 5% CO2. Transfections of cells were carried out using Lipofectamine™ 3000 Transfection Reagent (Invitrogen) according to the manufacturer’s instructions. Cells were harvested after 24 h transfection for subsequent treatments. The human MDK-mediated shRNA sequences were: Oligo Sequence 1 CCGGCAAGACCAAAGCAAAGGCCAACTCGAGTTGGCCTTTGCTTTGGTCTTGTTTTTG; Oligo Sequence 2 CCGGCGACTGCAAGTACAAGTTTGACTCGAGTCAAACTTGTACTTGCAGTCGTTTTTG.

### MTT assay

HNSCC cells were plated in a 96-well plate (3000 cells/well) and treated with or without irbesartan (IRB) at indicated dosages for 72 h. After treatment, 100 μl of 0.5 mg/ml MTT was added to each well and incubated at 37 °C for three hours. The medium was then removed, and 100 μl DMSO was added to each well to lyse cells. Plates were measured at 595 nm using Umax Kinetic Microplate Reader (Molecular Devices, Celifomie, USA). We purchased human MDK and IRB from Sigma (Sigma Aldrich, St Louis, MO, USA).

### Migration and invasion assay

Transwell inserts (pore size: 8 μm) coated with or without Matrigel (BD Biosciences) were used to evaluate the migratory and invasive abilities of CAL27, SAS and HSC-3 cells. For migration assays, 1 × 10^4^ cells in 100 μl of serum-free medium in the upper chamber and added 500 μl of medium in the lower chamber. For invasion assays, inserts coated 5% matrigel in PBS. 2 × 10^4^ cells were added in 100 μl of serum-free medium in the upper chamber and 500 μl of DMEM medium in the lower chamber. After the cells were incubated for 20 h, they were fixed and stained with crystal violet for 15 min. The numbers of migratory and invasive cells were counted in five fields under a microscope. All groups of experiments were conducted in triplicate.

### Statistical analysis

The SPSS 19 software was used to analyze the HNSCC patients’ data. To compare data between the two groups, we performed the Chi-square test and Fisher’s exact test. The Kaplan–Meier method was used for univariate analysis of DFS and OS, and a log-rank method tested the difference between survival curves. The significant parameters at the univariate level were assigned to the Cox regression model to analyze their relative prognostic importance. For HNSCC cell line experiments, a t-test was used for the statistical analysis. Every study was carried out independently at least twice, with three repeats each.

## Results

### Immunohistochemical expression of MDK and AT1R and its correlations with other clinicopathologic parameters in resectable HNSCC

The clinicopathologic factors of the 150 patients with HNSCC post-operation showed in Table 1. The median age of these patients was 51 years old (ranging from 29 to 75 years). One hundred and forty-two patients were male, and eight patients were female. The most common primary tumor site was oral (98 patients, 65.3%), followed by hypopharynx/larynx (28 patients, 18.7%) and oropharynx (24 patients, 16%). Before surgery, 55 patients (36.7%) were ECOG performance status 0, 68 patients (45.3%) were 1, and 27 patients (18%) were 2. The pathological AJCC tumor stage 1 accounted for eight patients (5.3%), stage II for 25 patients (16.7%), stage III for 26 patients (17.3%), and stage IVA-B for 91 patients (60.7%). The P16 positive rate was 12.7% (19 patients), and the extra-nodal extensions (ENE) rate was 30.7% (46 patients). Fifty-four patients (36%) had lymphovascular invasion (LVI), and 69 patients (46%) had perineural invasion (PNI). One hundred and twenty HNSCC patients in this study had a habit of cigarette smoking, 106 patients had alcohol consumption, and 99 patients had betel nuts chewing. Of these 150 HNSCC patients, 69 had medical records of diabetes mellitus, 50 had viral hepatitis, and 81 had hypertension.

**Table 1:** Clinical characteristics of HNSCC in this study

	Patient Numbers	Percentage (%)
Gender		
Male	142	94.7
Female	8	5.3
Age (Median: 51 ± 9.9 years old)		
≦51	76	50.7
> 51	74	49.3
Performance status (ECOG)		
0	55	36.7
1	68	45.3
2	27	18.0
Tumor site		
Oral	98	65.3
Oropharynx	24	16.0
Hypopharynx/larynx	28	18.7
Tumor stage		
T1	10	6.7
T2	48	32.0
T3	30	20.0
T4	62	41.3
Lymph nodes		
N0	63	42.0
N1	26	17.3
N2	56	37.3
N3	5	3.3
AJCCTMN stage		
I	8	5.3
II	25	16.7
III	26	17.3
IV	91	60.7
P16		
Positive	19	12.7
Negative	131	87.3
Extra-nodal extension		
Positive	46	30.7
Negative	104	69.3
Viral hepatitis		
No	100	66.6
Yes	50	33.3
Diabetes mellitus		
No	94	62.7
Yes	56	37.3
Hypertension		
No	69	46.0
Yes	81	54.0
Lyphovascular invasion		
No	96	64.0
Yes	54	36.0
Perineural invasion		
No	81	54.0
Yes	69	46.0
Adjuvant treatment		
No	38	25.3
RT	37	24.7
CCRT	75	50.0
Smoking		
No	30	20.0
Yes	120	80.0
Alcohol		
No	44	29.3
Yes	106	70.7
Betel nuts		
No	51	34.0
Yes	99	66.0
Midkine		
Weak	88	58.7
Strong	62	41.3
AT1R		
Weak	92	61.3
Strong	58	38.7

**Fig. 1:**
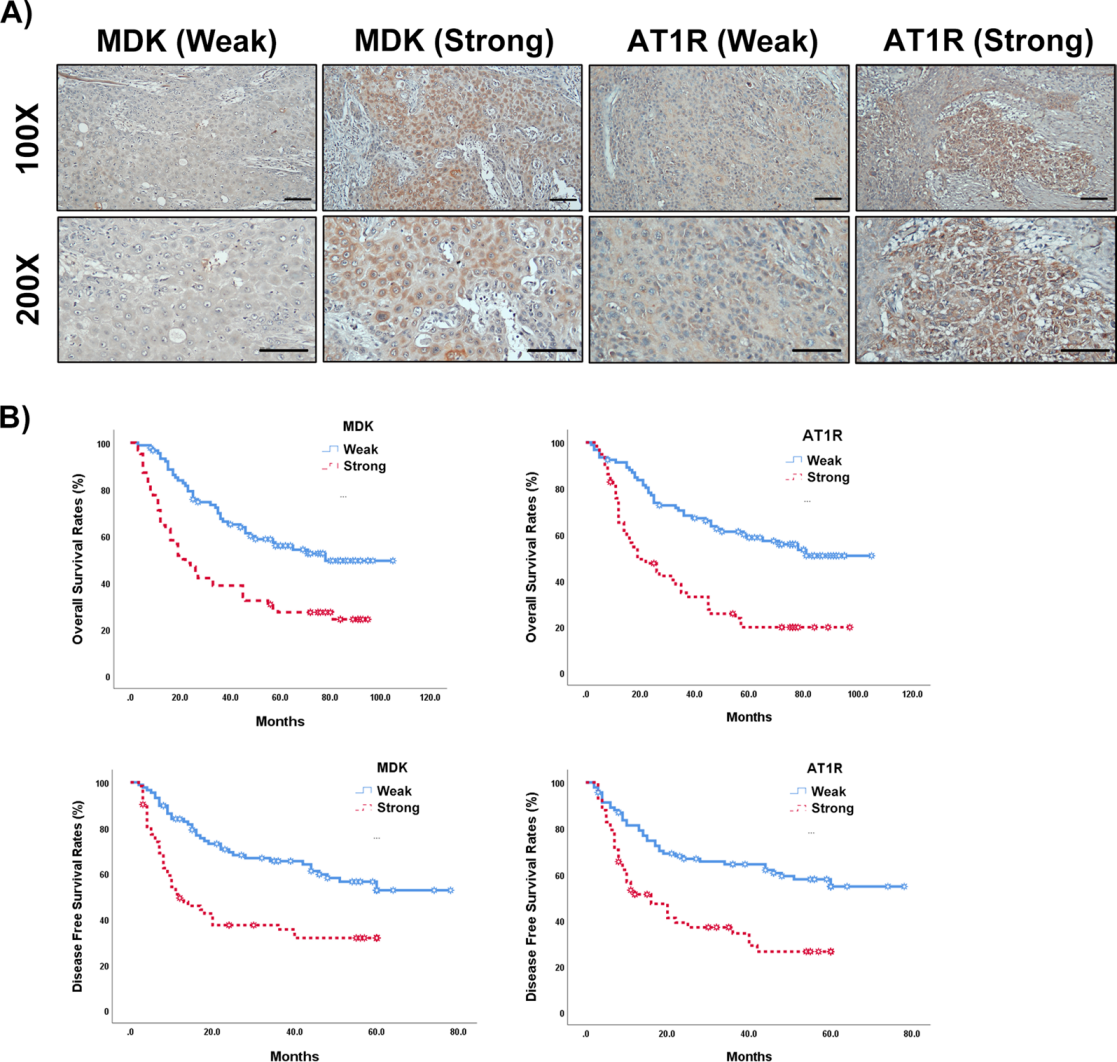
MDK and AT1R expressions are correlated with poor prognoses. **A** MDK, and AT1R expressions were evaluated by immunohistochemical staining (100× and 200×). Scale bar = 100 μm. **B** Positive MDK and AT1R expression were associated with poor DFS and OS by Kaplan–Meier analyses

Table 2 describes the correlation between the clinicopathological factors with immunohistochemical expression of AT1R and MDK (Fig. 1A). Strong AT1R expression was significantly associated with AJCC tumor stage (stage IVA/B, p = 0.001), hypertension (p = 0.004), and strong MDK expression (p < 0.001). Strong MDK expression was also associated with AJCC tumor stage (p = 0.004), lymph node metastases (p < 0.001), ENE (p < 0.001), and AT1R expression (p < 0.001).

**Table 2:** Correlation between expression of AT1R and MDK and clinicopathological factors of HNSCC

	No. of patients	AT1R		P	MDK		P
		Weak expression	Strong expression		Weak expression	Strong expression	
Age				0.226			0.599
≦51	76	43 (56.6%)	33 (43.4%)		43 (56.6%)	33 (43.4%)	
> 51	74	49 (66.2%)	25 (33.8%)		45 (60.8%)	29 (39.2%)	
Gender				0.944			0.141
Male	142	87 (61.3%)	55 (38.7%)		81 (57.0%)	61 (43.0%)	
Female	8	5 (62.5%)	3 (37.5%)		7 (87.5%)	1 (12.5%)	
ECOG PS				0.087			0.072
0	55	40 (72.7%)	15 (27.3%)		37 (67.3%)	18 (32.7%)	
1	68	38 (55.9%)	30 (44.1%)		40 (58.8%)	28 (41.2%)	
2	27	14 (51.9%)	13 (48.1%)		11 (40.7%)	16 (59.3%)	
Tumor site				0.182			0.249
Oral cavity	98	64 (65.3%)	34 (37.4%)		62 (63.3%)	36 (36.7%)	
Oropharynx	24	15 (62.5%)	9 (37.5%)		13 (54.2%)	11 (45.8%)	
Hypopharynx/larynx	28	13 (46.4%)	15 (53.6%)		13 (46.4%)	15 (53.6%)	
AJCC tumor stage				0.001^**^			0.004^**^
I–III	59	45 (76.3%)	14 (23.7%)		43 (72.9%)	16 (27.1%)	
IVA–B	91	47 (51.6%)	44 (48.4%)		45 (49.5%)	46 (50.5%)	
T stage				0.303			0.424
1–3	88	57(64.8%)	31(35.2%)		54 (61.4%)	34 (38.6%)	
4A–B	62	35 (56.5%)	27 (43.5%)		34 (54.8%)	28 (45.2%)	
N stage				0.423			< 0.001^**^
Negative	63	41(65.1%)	22(34.9%)		50 (79.4%)	13 (20.6%)	
Positive	87	51 (58,6%)	36 (41.4%)		38 (43.7%)	49 (56.3%)	
P16 expression				0.405			0.324
Negative	131	82 (62.6%)	49 (37.4%)		79 (60.3%)	52 (39.7%)	
Positive	19	10 (52.6%)	9 (47.4%)		9 (47.4%)	10 (52.6%)	
ENE				0.001^**^			< 0.001^**^
Negative	104	73 (70.2%)	31 (29.8%)		75 (72.1%)	29 (27.9%)	
Positive	46	19 (41.3%)	27 (58.7%)		13 (28.3%)	33 (71.7%)	
LVI 0.759							0.814
Negative	96	58 (60.4%)	38 (39.6%)		57 (59.4%)	39 (40.6%)	
Positive	54	34 (63.0%)	20 (37.0%)		31 (57.4%)	23 (42.6%)	
PNI 0.435							0.622
Negative	81	52 (64.2%)	29 (35.8%)		49 (60.5%)	32 (39.5%)	
Positive	69	40 (58.0%)	29 (42.0%)		39 (56.5%)	30 (43.5%)	
Alcohol drinking				0.065			0.127
No	44	32 (72.7%)	12 (27.3%)		30 (68.2%)	14 (31.8%)	
Yes	106	60 (56.6%)	46 (43.4%)		58 (54.7%)	48 (45.3%)	
Smoking				0.276			0.562
No	30	21 (70.0%)	9 (30.0%)		19 (63.3%)	11 (36.7%)	
Yes	120	71(59.2%)	49 (40.8%)		69 (57.5%)	51(42.5%)	
Betel nuts				0.651			0.075
No	51	30 (58.8%)	21 (41.2%)		35 (68.6%)	16 (31.4%)	
Yes	99	62 (62.6%)	37 (37.4%)		53 (53.5%)	46 (46.5%)	
DM				0.132			0.525
Negative	94	62 (66.0%)	32 (34.0%)		57 (60.6%)	37 (39.4%)	
Positive	56	30 (53.6%)	26 (46.4%)		31 (55.4%)	25 (44.6%)	
Hypertension				0.004^**^			0.242
No	69	52 (75.4%)	17 (24.6%)		44 (63.8%)	25 (36.2%)	
Yes	81	40 (49.4%)	41 (50.6%)		44 (54.3%)	37 (45.7%)	
Viral hepatitis				0.236			0.348
No	100	58 (58.0%)	42 (42.0%)		56 (56.0%)	44 (44.0%)	
Yes	50	34 (68.0%)	16 (32.0%)		32 (64.0%)	18 (36.0%)	
MDK				< 0.001**			
Weak	88	66 (75.0%)	22 (25.0%)				
Strong	62	26 (41.9%)	36 (58.1%)				
AT1R							< 0.001**
Weak	92				67 (72.8%)	25 (27.2%)	
Strong	58				21 (36.2%)	37 (63.8%)	

Bold indicates statistically significant results

### Survival analysis of HNSCC after tumor resection

The median follow-up duration of this study was 44 months (ranging from 2 to 105 months). After tumor resection, 78 patients experienced tumor recurrence, and 86 died during this period. The median DFS time was 26 months, and the 2-years DFS rate was 56.2%. The median OS duration was 46 months, and the 5-year OS rate was 43.6%. The univariate analyses demonstrated that ECOG performance status 1 or 2 (p < 0.001), T stage 4A/4B (p = 0.001), lymph node metastases (p = 0.005), AJCC tumor stage IVA/IVB (p < 0.001), positive LVI (p = 0.005, positive PNI (p < 0.001), positive extra-nodal extension (p < 0.001), the habitus of betel nuts chewing (p = 0.006), strong MDK (p = 0.001, Fig. 1B), and strong AT1R expression (p = 0.009, Fig. 1B) had poor shorter DFS (Table 3). In addition, our study also indicated that ECOG PS 0/1 (p < 0.001), T stage 4A/4B (p = 0.016), lymph nodes metastases (p = 0.003), AJCC tumor stage IVA/B (p < 0.001), positive LVI (p = 0.001), positive PNI (p < 0.001), ENE (p < 0.001), P16 positive (p = 0.015), cigarette smoking (p = 0.029), betel nuts chewing (p = 0.006), viral hepatitis (p = 0.017), strong AT1R(p = 0.001, Fig. 1E), and strong MDK expression (p < 0.001, Fig. 1) were associated with inferior OS.

**Table 3:** Correlation between the clinicopathological features and Disease-Free survival in HNSCC

Variable	Category	Univariate analysis			Multivariate analysis		
		HR	95% CI	p	Adjusted HR	95% CI	p
Age	≦51 vs > 51	0.932	0.59–1.43	0.724	0.742	0.46–1.21	0.228
Gender	Female vs male	0.814	0.30–2.23	0.689	0.871	0.28–2.69	0.810
Tumor site	Oral	Reference			Reference		
	Oropharynx	0.609	0.30–1.23	0.168	0.715	0.33–1.53	0.387
	Hypopharynx/larynx	0.931	0.53–1.65	0.806	0.477	0.28–0.98	**0.046**
ECOG PS	0	Reference			Reference		
	1	4.129	2.23–7.63	** < 0.001**	3.07	1.50–6.26	**0.002**
	2	18.296	8.88–37.70	** < 0.001**	9.478	3.90–23.06	**0.001**
T stage	1–3 vs 4A–B	0.478	0.25–0.71	**0.001**			
N stage	Negative vs positive	0.515	0.32–0.83	**0.005**			
AJCC stage	I–III vs IVA–B	0.404	0.25–0.67	** < 0.001**	0.917	0.51–1.64	0.770
LVI	Negative vs positive	0.521	0.33–0.82	**0.005**	0.703	0.42–1.17	0.177
PNI	Negative vs positive	0.326	0.21–0.52	** < 0.001**	0.507	0.29–0.88	**0.017**
ENE	Negative vs Positive	0.221	0.14–0.35	** < 0.001**	0.298	0.15–0.58	**0.001**
P16 expression	Negative vs positive	1.725	0.83–3.59	0.145	1.385	0.61–3.16	0.438
Alcohol drinking	No vs yes	1.050	0.65–1.70	0.844	0.890	0.52–1.54	0.676
Smoking	No vs yes	0.742	0.42–1.32	0.313	1.222	0.67–2.45	0.519
Betel nuts	No vs yes	0.426	0.25–0.72	**0.002**	0.555	0.32–0.98	**0.041**
DM	No vs yes	1.005	0.63–1.40	0.983	1.228	0.73–2.07	0.439
Hypertension	No vs yes	1.047	0.67–1.63	0.841	1.227	0.74–2.03	0.425
Viral hepatitis	No vs yes	0.838	0.53–1.34	0.458	1.310	0.74–2.33	0.358
MDK	Weak vs strong	0.446	0.29–0.70	**0.001**	0.312	0.15–0.66	**0.038**
AT1R	Weak vs strong	0.552	0.35–0.86	**0.009**	0.755	0.44–1.29	0.303

Bold indicates statistically significant results

In a multivariate comparison, MDK expression remained independently associated with DFS, together with ECOG PS, PNI, ENE, and betel nuts chewing (Table 3). For OS, multivariate Cox regression analysis also showed that MDK, ECOG PS, LVI, and ENE were independent prognostic factors (Table 4).

**Table 4:** Overall survival associated with clinicopathological factors in post-operation HNSCC patients

Variable	Category	Univariate analysis			Multivariate analysis		
		HR	95% CI	p	Adjusted HR	95% CI	p
Age	≦51 vs > 51	1.008	0.66–1.54	0.970	0.683	0.43–1.10	0.113
Gender	Female vs male	0.305	0.08–1.24	0.097	0.510	0.12–2.25	0.373
Tumor site	Oral	Reference			Reference		
	Oropharynx	1.023	0.59–1.78	0.935	0.648	0.31–1.36	0.253
	Hypopharynx/larynx	0.610	0.28–1.35	0.221	0.477	0.24–0.95	**0.034**
ECOG PS	0	Reference			Reference		
	1	4.355	2.42–7.83	** < 0.001**	2.81	1.42–5.60	**0.003**
	2	17.229	8.73–34.00	** < 0.001**	7.662	3.16–18.58	** < 0.001**
T stage	0–3 vs 4A–B	0.594	0.39–0.91	**0.016**			
N stage	Negative vs positive	0.507	0.32–0.80	**0.003**			
AJCC stage	I–III vs IVA–B	0.411	0.26–0.66	** < 0.001**	1.035	0.59–1.83	0.905
LVI	Negative vs positive	0.469	0.31–0.72	**0.001**	0.449	0.28–0.72	**0.001**
PNI	Negative vs positive	0.279	0.18–0.44	** < 0.001**	0.608	0.35–1.06	0.077
ENE	Negative vs positive	0.179	0.11–0.0.28	** < 0.001**	0.241	0.13–0.46	** < 0.001**
P16 expression	Negaive vs positive	2.814	1.23–6.46	**0.015**	2.452	0.98–6.13	0.055
Alcohol drinking	No vs yes	1.031	0.65–1.64	0.896	0.850	0.51–1.42	0.535
Smoking	No vs yes	0.494	0.26–0.93	**0.029**	0.862	0.44–1.68	0.519
Betel nuts	No vs yes	0.506	0.31–0.82	**0.006**	0.701	0.41–1.19	0.188
DM	No vs yes	0.754	0.49–1.16	0.196	1.093	0.68–1.75	0.712
Hypertension	No vs yes	0.794	0.52–1.22	0.289	0.736	0.45–1.20	0.216
Viral hepatitis	No vs yes	0.590	0.38–0.91	**0.017**	0.711	0.40–1.26	0.240
MDK	Weak vs strong	0.433	0.28–0.66	** < 0.001**	0.475	0.28–0.82	**0.007**
AT1R	Weak vs strong	0.472	0.31–0.72	**0.001**	0.597	0.35–1.03	0.065

Bold indicates statistically significant results

### Irbesartan (IRB) inhibited HNSCC cell proliferation

**Fig. 2:**
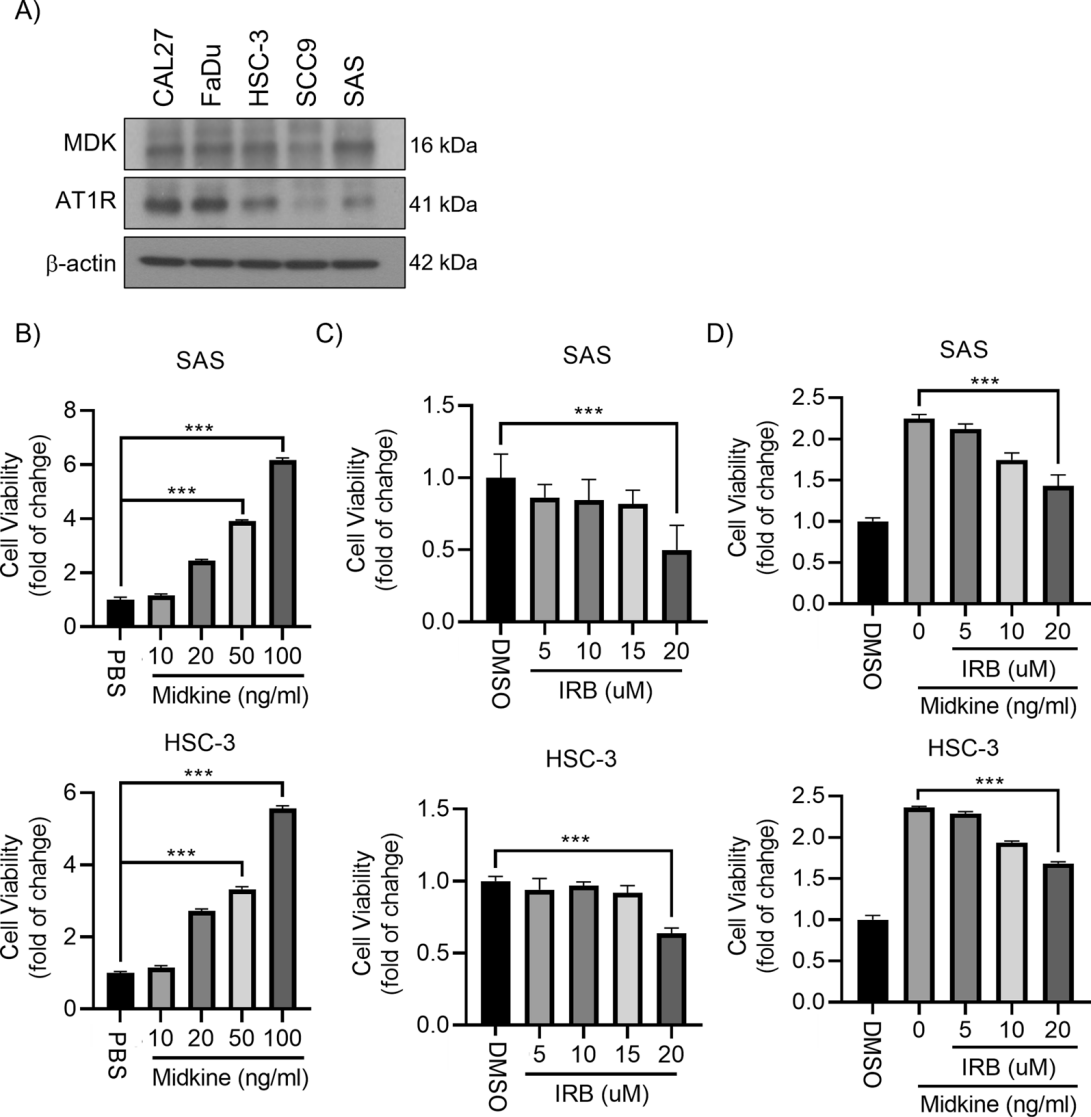
MDK and AT1R were expressed in HNSCC cell lines. **A** Western blots showed MDK and AT1R protein expressions in human HNSCC cell lines. **B**–**D** MTT assay showed MDK increased HNSCC cell proliferation (**B**). IRB decreased HNSCC cell proliferation (**C**). IRB suppressed MDK-induced HNSCC cell proliferation (**D**). *p < 0.05; **p < 0.01; ***p < 0.001. Data are presented as mean ± SD

Irbesartan (IRB), an angiotensin II inhibitor and oral selective AT1R blocker, extensively treats high blood pressure. We initially surveyed the protein expressions of the MDK and AT1R by Western blotting in 5 HNSCC cell lines and confirmed both proteins were expressed in these cell lines, including HSC-3 and SAS (Fig. 2A). In SAS and HSC-3 cell lines, MDK could stimulate cell proliferation (Fig. 2B), and IRB could suppress HNSCC cells (Fig. 2C). In addition, IRB also inhibited the MDK-dependent growth of HNSCC cells (Fig. 2D).

### MDK regulated AT1R expression and HNSCC cell growth and motility

**Fig. 3:**
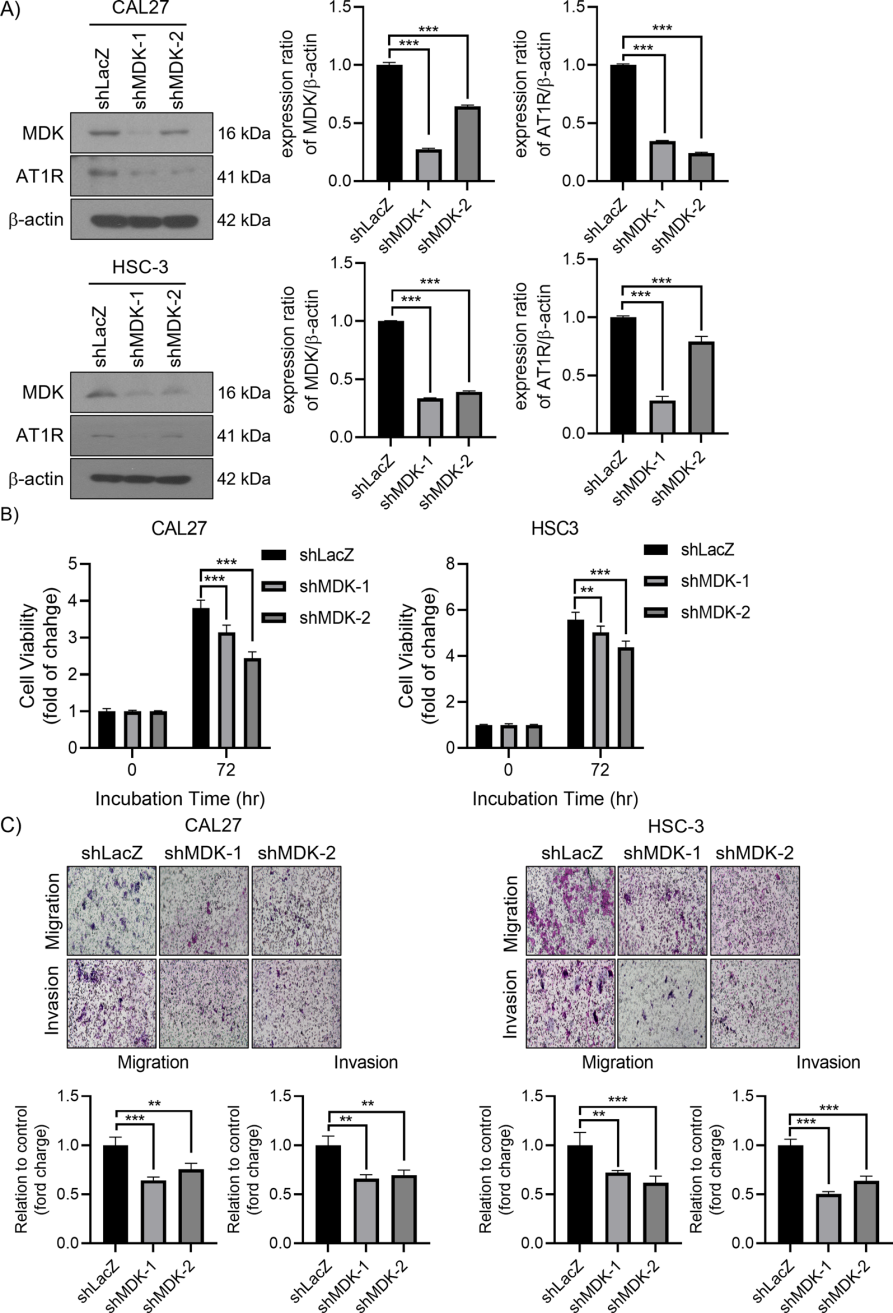
Suppressed MDK reduced AT1R expression, proliferation, invasion, and migration in the HNSCC cells. **A** Protein expressions of MDK and AT1R were evaluated in CAL27 and HSC-3 cells by Western blotting after cells were transfected with MDK shRNA and control shRNA, shLacZ. **B** MTT tests were performed to estimate the cell proliferation of CAL27 and HSC-3 cells transfected with shMDK and shLacZ. **C** Transwell tests were performed to assess the motility of CAL27 and HSC-3 cells transfected with shMDK and shLacZ *p < 0.05; **p < 0.01; ***p < 0.001. Data are presented as mean ± SD

Transfected with MDK shRNA or shControl in CAL27 and HSC-3 were analyzed by Western blotting (Fig. 3A). After being transfected with shMDK, the AT1R expression was significantly reduced (Fig. 3A). The MTT tests showed that depleted MDK significantly suppressed the cell proliferation of CAL27 and HSC-3 when compared with the shControls (Fig. 3B). We also performed invasion and migration assays in MDK-suppressed CAL27 and HSC-3 cells. As shown in Fig. 3C, the knock-down of MDK significantly inhibited cell migration and invasion compared to control.

**Fig. 4:**
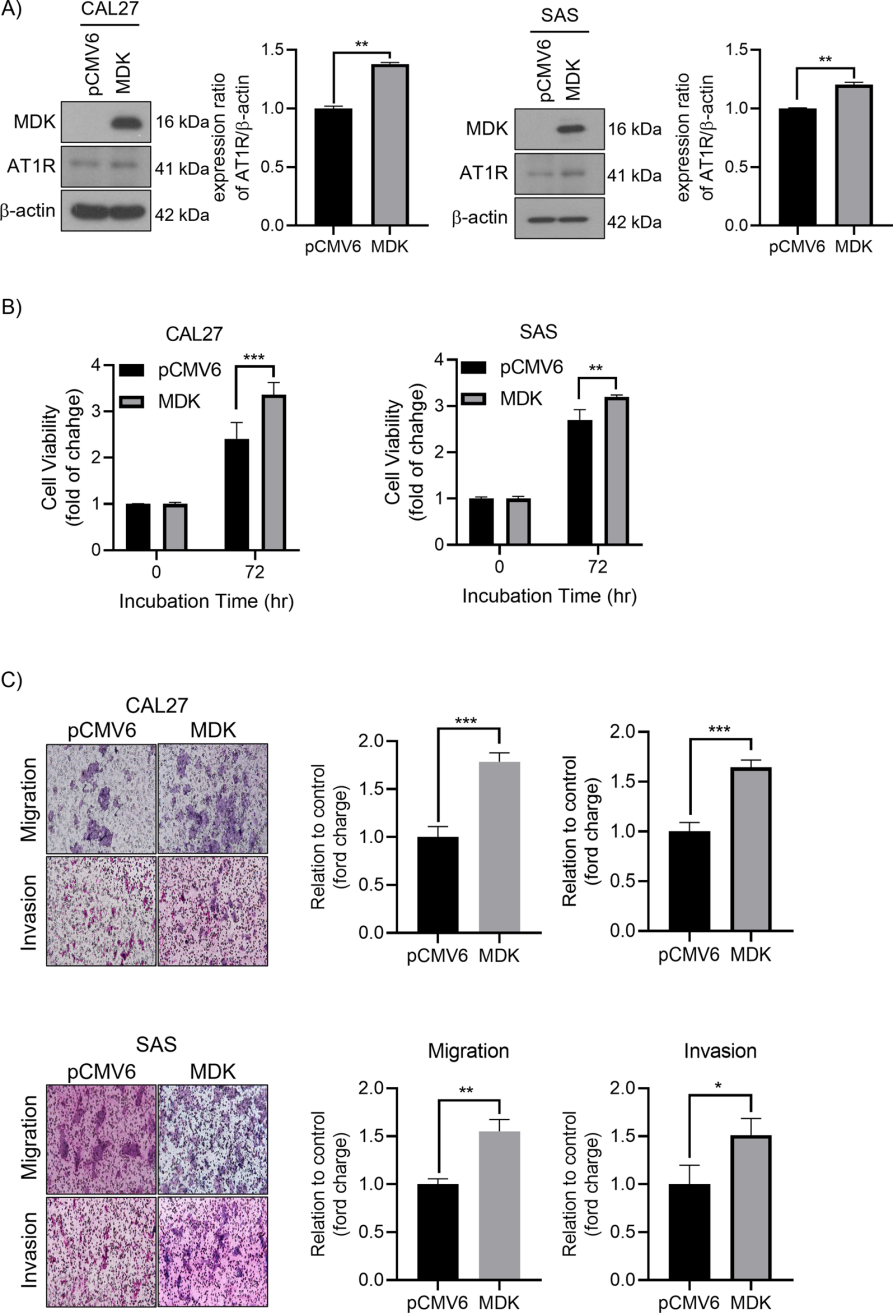
Overexpressed MDK increased AT1R expression, proliferation, and growth in HNSCC cell lines. **A** The protein levels of MDK and AT1R were evaluated by western blotting in CAL27 and SAS cells with MDK overexpressed. **B** MTT tests showed cell growth in the CAL27 and SAS cells treansfected with MDK overexpressed was significantly elevated when compared to the cells transfected with pCMV6-control. **C** Represented figure (left) and quantification result (right) showed the cell motility was increased in the cells with MDK overexpression. *p < 0.05; **p < 0.01; ***p < 0.001. Data are presented as mean ± SD

Overexpressed MDK in the HNSCC cell lines, CAL27 and SAS, promoted AT1R expressions (Fig. 4A). The MTT test also showed increased HNSCC cell lines proliferation while MDK overexpressed (Fig. 4B), and the migration and invasion increased in Transwell assays (Fig. 4C).

### MDK also affected pAkt expression in human HNSCC cells

**Fig. 5:**
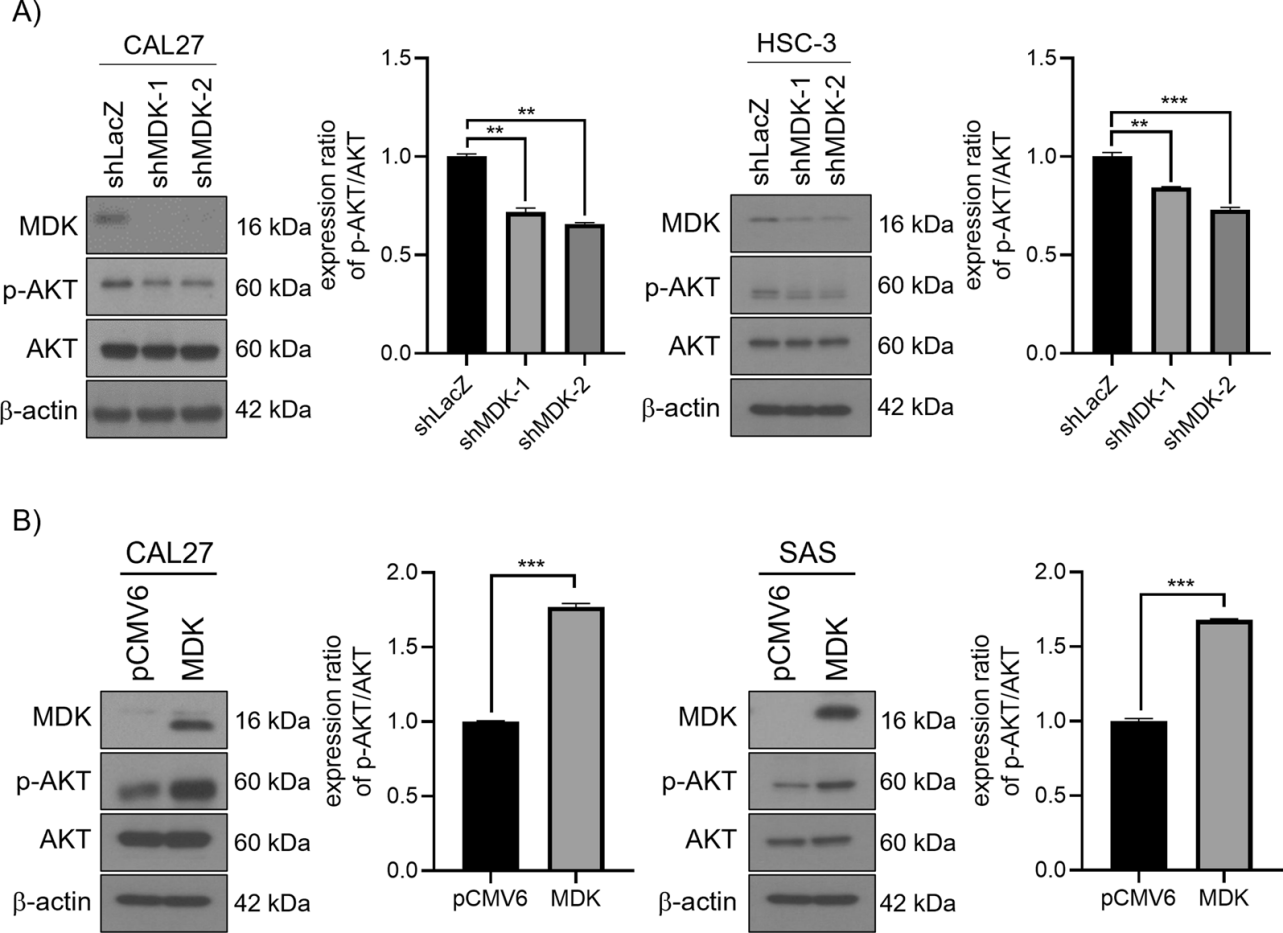
MDK regulated pAKT expression in HNSCC cell lines. **A** Western blotting were used to evaluate pAKT expression. Compared with shLAcZ, pAKT has significantly been suppressed in shMDK-transfected CAL27 and HSC-3 cell lines. **B** Western blotting showed that pAKT expression was increased in the SAS and CAL-27 cell lines with MDK overexpression. *p < 0.05; **p < 0.01; ***p < 0.001. Data are presented as mean ± SD

As shown in Fig. 5A, pAKT expression was suppressed while knocked down MDK in CAL27 and HSC-3 cells. In contrast, pAKT expression was increased while MDK was overexpressed in HNSCC cell lines SAS and CAL27 (Fig. 5B).

## Discussion

Our study showed a potentially new mechanism to improve survival in resectable HNSCC patients. AT1R and MDK expression was significantly correlated in HNSCC patient tissue samples. The positive expression of MDK and AT1R in HNSCC patients predicted poor DFS and OS. Silencing MDK in HNSCC cells decreased their proliferation, invasion, and migration. Inhibition of MDK also suppressed AT1R and p-AKT expression in our HNSCC cell lines. IRB could suppress the MDK-stimulating HNSCC cell growth. These findings suggested that AT1R could be targeted in the MDK-positive HNSCC.

Complete tumor resection is the mainstay of curative treatment of early and localized advanced HNSCC. Tumor recurrence often impacts patients’ survival due to lower remission and higher mortality. Adjuvant radiotherapy or concurrent chemoradiotherapy after operation in the pathological high-risk group is the standard management. High locoregional failure and poor disease-free duration suggested lacking effective therapy and even received maintenance treatments [15–17]. Several studies have demonstrated that MDK is an effective biomarker for predicting the outcomes of HNSCC patients [18]. Our previous study also found that MDK expression was associated with lower disease-free and OS rates after surgery. Our current study also consistently results with positive MDK expression in HNSCC patients with prompt tumor recurrence. From the literature review, MDK seems a drugable target in different cancers [19], including oral squamous cell carcinoma [20]. However, no medication was available to block MDK expression directly in cancer patients. Our study provided inhibition of AT1R by IRB may reduce tumor progression in MDK-expressed HNSCC patients.

Growth factors promote cancer cell proliferation, invasion, and migration. MDK activates the AKT pathway to promote GBM and oral squamous cell carcinoma progression [20, 21]. In the current study, MDK expression was associated with advanced tumor stage, lymph node metastases, and extra-nodal extension. These findings demonstrated our previous study results and predict HNSCC patients’ poor prognoses. MDK is a secreted protein, and the concentration of MDK increased significantly in the MDK overexpressed HNSCC medium (Additional file 1: Fig. S1). However, the secreted MDK-affected HNSCC cell function mechanism needs more well-designed research. Currently, a novel finding in our study was that MDK and AT1R expression was highly correlated. All uncropped western blotting membranes were showed in Additional file 2.

Limited research explored the relationship between MDK and RAS in cancers. Akinori et al. showed that MDK protein enhanced ACE expression in mice with chronic kidney disease [10]. Their study showed that nephrectomy-induced MDK expression increases ACE activity and plasma angiotensin II levels. To our best knowledge, there is no literature to discuss how MDK affects AT1R expression in cancer. Our current study showed that MDK and AT1R expression was highly correlated in HNSCC patients, and MDK could regulate AT1R expression in HNSCC cells. However, there was no interaction between MDK and AT1R in HNSCC cells by Co-IP approach (data not shown), indicating that MDK might modulate the AT1R protein stability via other pathways, such as proteasome pathway or ubiquitin pathway. Further experiments will be designed and performed in the future. The AT1R and pAKT expressions were also down-regulated while shMDK was transfected into HNSCC cell lines. In contrast, the AT1R and pAKT upregulated while MDK was overexpressed.

Increasing evidence show that AT1R is involved in tumor growth, metastases, and angiogenesis in different animal models [22]. ACE synthesizes angiotensin II and stimulates tumor cell growth through AT1R. Selective AT1R blockade might be more effective than ACE inhibition [23]. Although recent research illustrated the activation of RAS and upregulation of AT1R in different tumor tissues [24, 25], there were no reports to analyze the AT1R expression in HNSCC. Our current study also showed AT1R was associated with advanced tumor stage, hypertension, MDK expression, and worse survival in HNSCC patients. IRB could inhibit HNSCC cell growth by suppressing AT1R under MDK stimulation. AT1R may play an important role in MDK enhancing HNSCC cell proliferation. This result could explain oral squamous cell carcinoma patients who received ARB improved overall survival in our retrospective study. Lin et al. also showed ARB had effects of anti-proliferation and anti-angiogenesis in nasopharyngeal cancer patients [26].

One study in breast cancer also found AT1R increases cell migration through the AKT pathway [27]. Recently, Zhang et al. reported that suppression of AT1R expression inhibited lung cancer cell proliferation and migration by regulating the AKT pathway [28]. It has been observed that either AT1R or MDK can activate the AKT pathway. However, it has not been shown whether MDK interacts with AT1R to impact the AKT signaling pathway involved in driving HNSCC cell viability, growth, and motility. In our study, we found that knockdown of MDK resulted in a reduction in the expression of both AT1R and pAkt. Furthermore, we also showed that the activity or function of MDK in promoting cell viability is dependent on the presence or activation of AT1R. These findings suggest that MDK modulates the RAS pathway through AT1R. In sum, these findings highlight the potential interaction between MDK, AT1R, and the pAkt signaling pathway, which appears to be involved in HNSCC cell viability, growth, and motility. The current study’s limitations included a retrospective study to enroll post-operative HNSCC patients. First, we wanted to evaluate the expressions of MDK and AT1R to affect DFS and OS in HNSCC patients post-operation. However, in HNSCC, the second primary tumor in a different location and repeated tumor resection may affect DFS and OS. Besides, most of our HNSCC patients were male (94.7%) and came from the oral cavity. In Taiwan, smoking, alcohol, and betel nuts are the essential risk factors for HNSCC patients, and most are male. Second, our study found that IRB could inhibit HNSCC cell proliferation, even in MDK stimulation. This finding needs carcinogen-induced HNSCC mouse models or xenograft models to help verification in the future. Third, our study found that MDK influences AT1R expression and affects proliferation, migration, and invasion in HNSCC cells. However, the mechanism of MDK regulating AT1R to control HNSCC cell functions is unclear.

## Conclusion

Our study showed MDK and AT1R were important prognostic factors in resectable HNSCC patients. MDK and AT1R were highly correlated, and MDK affected AT1R and pAKT expressions in HNSCC. Suppression of AT1R by IRB decreased HNSCC cell proliferation even under MDK stimulation. Overall, these findings underscore the importance of the interplay between MDK, AT1R, and the pAkt signaling pathways in driving HNSCC cell viability, growth, and motility. More importantly, the blocking the AT1R pathway, possibly in combination with targeting MDK, could be a promising approach for the treatment of HNSCC.

### Supplementary Information

**Additional file 1: Figure S1.** MDK regulates AT1R and AT2R expression in HNSCC cell lines. **A **AT1R and AT2R expressions significantly increased while MDD overexpressed in the NHSCC cell line CAL27. **B** While we knocked down MDK with shMDK1 and shMDK2 in CAL27, AT1R and AT2R expressions also decreased. **C** Secreted MDK significantly increased in the medium of MDK overexpressed cells compared to the medium of control (pCMV6) cells.

**Additional file 2: Figure** **S2.** The complete image of the Western blot for the Figs. 2–5. **A** The uncropped blot for Fig. 2A.** B** The uncropped blot for Fig. 3A. **C** The uncropped blot for Fig. 4A. **D** The uncropped blot for Fig. 5A. **E** the uncropped blot for Fig. 5B.

## Data Availability

All data generated in this study are available in this article.

## References

[1] Mody MD, Rocco JW, Yom SS, Haddad RI, Saba NF (2021). Head and neck cancer. Lancet.

[2] Afsar B, Afsar RE, Ertuglu LA, Kuwabara M, Ortiz A, Covic A, Kanbay M (2021). Renin-angiotensin system and cancer: epidemiology, cell signaling, genetics and epigenetics. Clin Transl Oncol.

[3] Wegman-Ostrosky T, Soto-Reyes E, Vidal-Millan S, Sanchez-Corona J (2015). The renin-angiotensin system meets the hallmarks of cancer. J Renin Angiotensin Aldosterone Syst.

[4] Rosenthal T, Gavras I (2009). Angiotensin inhibition and malignancies: a review. J Hum Hypertens.

[5] Wu CN, Wu SC, Chen WC, Yang YH, Chin JC, Chien CY, Fang FM, Li SH, Luo SD, Chiu TJ (2021). Angiotensin II receptor blockers and oral squamous cell carcinoma survival: a propensity-score-matched cohort study. PLoS ONE.

[6] Chiu TJ, Chen YJ, Rau KM, Chen CH, Chien CY, Li SH, Tsai HT, Eng HL (2013). Midkine neurite growth-promoting factor 2 expression as a potential prognostic marker of adjuvant therapy in head and neck squamous cell carcinoma. Biomarkers.

[7] Erguven M, Bilir A, Yazihan N, Ermis E, Sabanci A, Aktas E, Aras Y, Alpman V (2011). Decreased therapeutic effects of noscapine combined with imatinib mesylate on human glioblastoma in vitro and the effect of midkine. Cancer Cell Int.

[8] Kadomatsu K, Kishida S, Tsubota S (2013). The heparin-binding growth factor midkine: the biological activities and candidate receptors. J Biochem.

[9] Muramatsu T (2014). Structure and function of midkine as the basis of its pharmacological effects. Br J Pharmacol.

[10] Hobo A, Yuzawa Y, Kosugi T, Kato N, Asai N, Sato W, Maruyama S, Ito Y, Kobori H, Ikematsu S (2009). The growth factor midkine regulates the renin-angiotensin system in mice. J Clin Invest.

[11] Li SH, Lu HI, Chang AY, Huang WT, Lin WC, Lee CC, Tien WY, Lan YC, Tsai HT, Chen CH (2016). Angiotensin II type I receptor (AT1R) is an independent prognosticator of esophageal squamous cell carcinoma and promotes cells proliferation via mTOR activation. Oncotarget.

[12] Arrieta O, Pineda-Olvera B, Guevara-Salazar P, Hernandez-Pedro N, Morales-Espinosa D, Ceron-Lizarraga TL, Gonzalez-De la Rosa CH, Rembao D, Segura-Pacheco B, Sotelo J (2008). Expression of AT1 and AT2 angiotensin receptors in astrocytomas is associated with poor prognosis. Br J Cancer.

[13] Ota K, Fujimori H, Ueda M, Jono H, Shinriki S, Ota T, Sueyoshi T, Taura M, Taguma A, Kai H (2010). Midkine expression is correlated with an adverse prognosis and is down-regulated by p53 in oral squamous cell carcinoma. Int J Oncol.

[14] Takeda H, Kondo S (2001). Differences between squamous cell carcinoma and keratoacanthoma in angiotensin type-1 receptor expression. Am J Pathol.

[15] Harrington K, Temam S, Mehanna H, D'Cruz A, Jain M, D'Onofrio I, Manikhas G, Horvath Z, Sun Y, Dietzsch S (2015). Postoperative adjuvant lapatinib and concurrent chemoradiotherapy followed by maintenance lapatinib monotherapy in high-risk patients with resected squamous cell carcinoma of the head and neck: a phase III, randomized, double-blind, Placebo-Controlled Study. J Clin Oncol.

[16] Matuschek C, Bolke E, Belka C, Ganswindt U, Henke M, Stegmaier P, Bamberg M, Welz S, Debus J, Gioules A (2013). Feasibility of 6-month maintenance cetuximab after adjuvant concurrent chemoradiation plus cetuximab in squamous cell carcinoma of the head and neck. Strahlenther Onkol.

[17] Racadot S, Thennevet I, Ouldbey Y, Kaminsky MC, Bosset M, Martin L, Tao Y, Sire C, de Raucourt D, Alfonsi M (2023). Afatinib maintenance therapy following post-operative radiochemotherapy in head and neck squamous cell carcinoma: results from the phase III randomised double-blind placebo-controlled study BIB2992ORL (GORTEC 2010–02). Eur J Cancer.

[18] Yamashita T, Shimada H, Tanaka S, Araki K, Tomifuji M, Mizokami D, Tanaka N, Kamide D, Miyagawa Y, Suzuki H (2016). Serum midkine as a biomarker for malignancy, prognosis, and chemosensitivity in head and neck squamous cell carcinoma. Cancer Med.

[19] Erdogan S, Doganlar ZB, Doganlar O, Turkekul K, Serttas R (2017). Inhibition of midkine suppresses prostate cancer CD133(+) stem cell growth and migration. Am J Med Sci.

[20] Masui M, Okui T, Shimo T, Takabatake K, Fukazawa T, Matsumoto K, Kurio N, Ibaragi S, Naomoto Y, Nagatsuka H (2016). Novel midkine inhibitor iMDK inhibits tumor growth and angiogenesis in oral squamous cell carcinoma. Anticancer Res.

[21] Hu B, Qin C, Li L, Wei L, Mo X, Fan H, Lei Y, Wei F, Zou D (2021). Midkine promotes glioblastoma progression via PI3K-Akt signaling. Cancer Cell Int.

[22] Egami K, Murohara T, Shimada T, Sasaki K, Shintani S, Sugaya T, Ishii M, Akagi T, Ikeda H, Matsuishi T (2003). Role of host angiotensin II type 1 receptor in tumor angiogenesis and growth. J Clin Invest.

[23] Fujimoto Y, Sasaki T, Tsuchida A, Chayama K (2001). Angiotensin II type 1 receptor expression in human pancreatic cancer and growth inhibition by angiotensin II type 1 receptor antagonist. FEBS Lett.

[24] Deshayes F, Nahmias C (2005). Angiotensin receptors: a new role in cancer?. Trends Endocrinol Metab.

[25] O'Rawe M, Kilmister EJ, Mantamadiotis T, Kaye AH, Tan ST, Wickremesekera AC (2021). The renin-angiotensin system in the tumor microenvironment of glioblastoma. Cancers (Basel)..

[26] Lin YT, Wang HC, Tsai MH, Su YY, Yang MY, Chien CY (2021). Angiotensin II receptor blockers valsartan and losartan improve survival rate clinically and suppress tumor growth via apoptosis related to PI3K/AKT signaling in nasopharyngeal carcinoma. Cancer.

[27] Zhao Y, Wang H, Li X, Cao M, Lu H, Meng Q, Pang H, Li H, Nadolny C, Dong X (2014). Ang II-AT1R increases cell migration through PI3K/AKT and NF-kappaB pathways in breast cancer. J Cell Physiol.

[28] Zhang S, Wang Y (2018). Telmisartan inhibits NSCLC A549 cell proliferation and migration by regulating the PI3K/AKT signaling pathway. Oncol Lett.

