# Supplementary material for: Prognosis of Midkine and AT1R expression in resectable head and neck squamous cell carcinoma
Source: Cancer Cell Int. 2023 Sep 24;23:212. doi: 10.1186/s12935-023-03060-z (PMC10518915; doi:10.1186/s12935-023-03060-z)
Supplement: Supplementary file 1 — Additional file 1: Figure S1. MDK regulates AT1R and AT2R expression in HNSCC cell lines. A AT1R and AT2R expressions significantly increased while MDD overexpressed in the NHSCC cell line CAL27. B While we knocked down MDK with shMDK1 and shMDK2 in CAL27, AT1R and AT2R expressions also decreased. C Secreted MDK significantly increased in the medium of MDK overexpressed cells compared to the medium of control (pCMV6) cells. [file 12935_2023_3060_MOESM1_ESM.docx]

**Additional file 1**


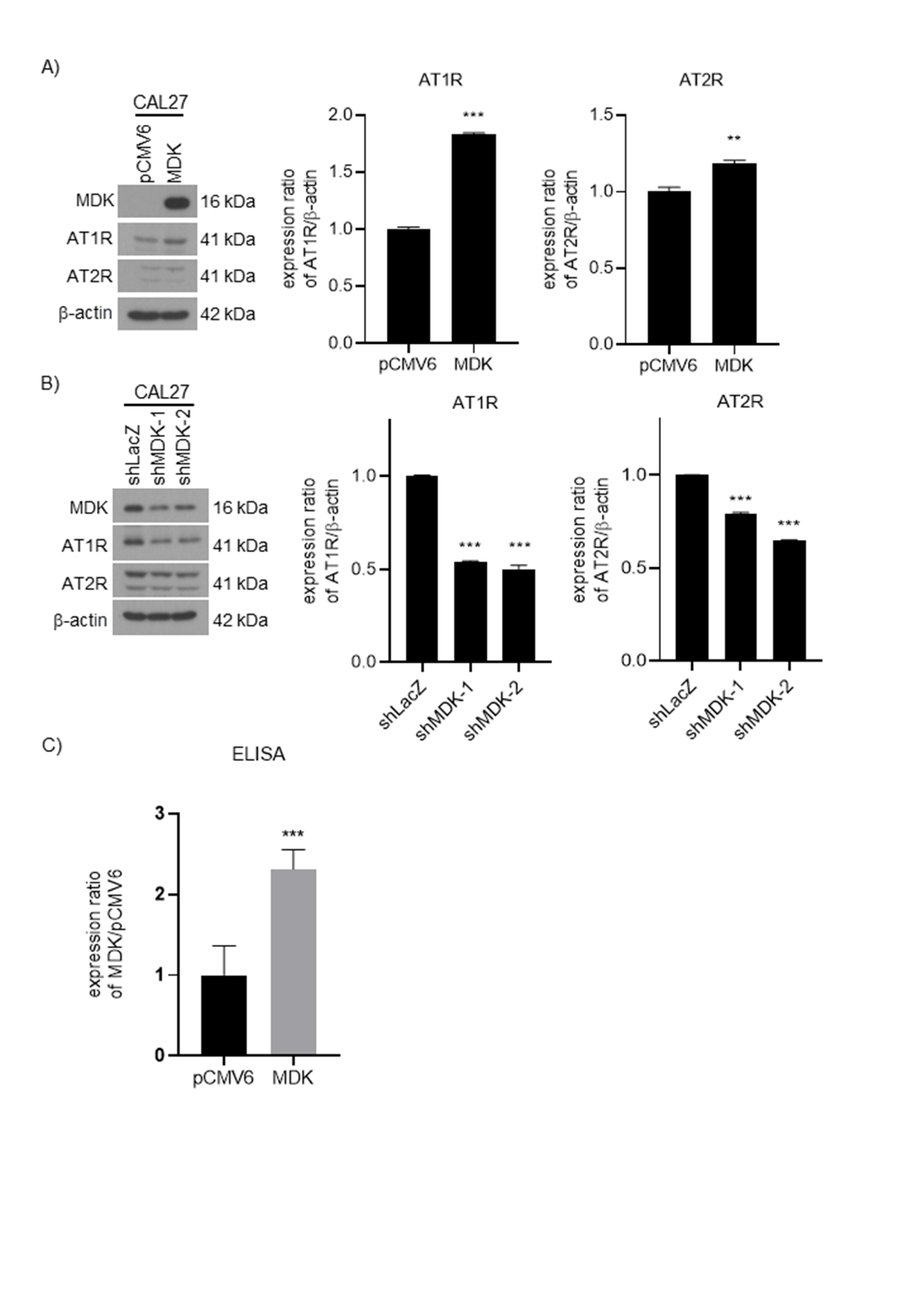


**Figure S1.** MDK regulates AT1R and AT2R expression in HNSCC cell lines. **A.** AT1R and AT2R expressions significantly increased while MDD overexpressed in the NHSCC cell line CAL27. **B.** While we knocked down MDK with shMDK1 and shMDK2 in CAL27, AT1R and AT2R expressions also decreased. **C.** Secreted MDK significantly increased in the medium of MDK overexpressed cells compared to the medium of control (pCMV6) cells.
