# Supplementary material for: Prognosis of Midkine and AT1R expression in resectable head and neck squamous cell carcinoma
Source: Cancer Cell Int. 2023 Sep 24;23:212. doi: 10.1186/s12935-023-03060-z (PMC10518915; doi:10.1186/s12935-023-03060-z)
Supplement: Supplementary file 2 — Additional file 2: Figure S2. The complete image of the Western blot for the Figs. 2–5. A The uncropped blot for Fig. 2A. B The uncropped blot for Fig. 3A. C The uncropped blot for Fig. 4A. D The uncropped blot for Fig. 5A. E the uncropped blot for Fig. 5B. [file 12935_2023_3060_MOESM2_ESM.docx]

**Additional file 2**


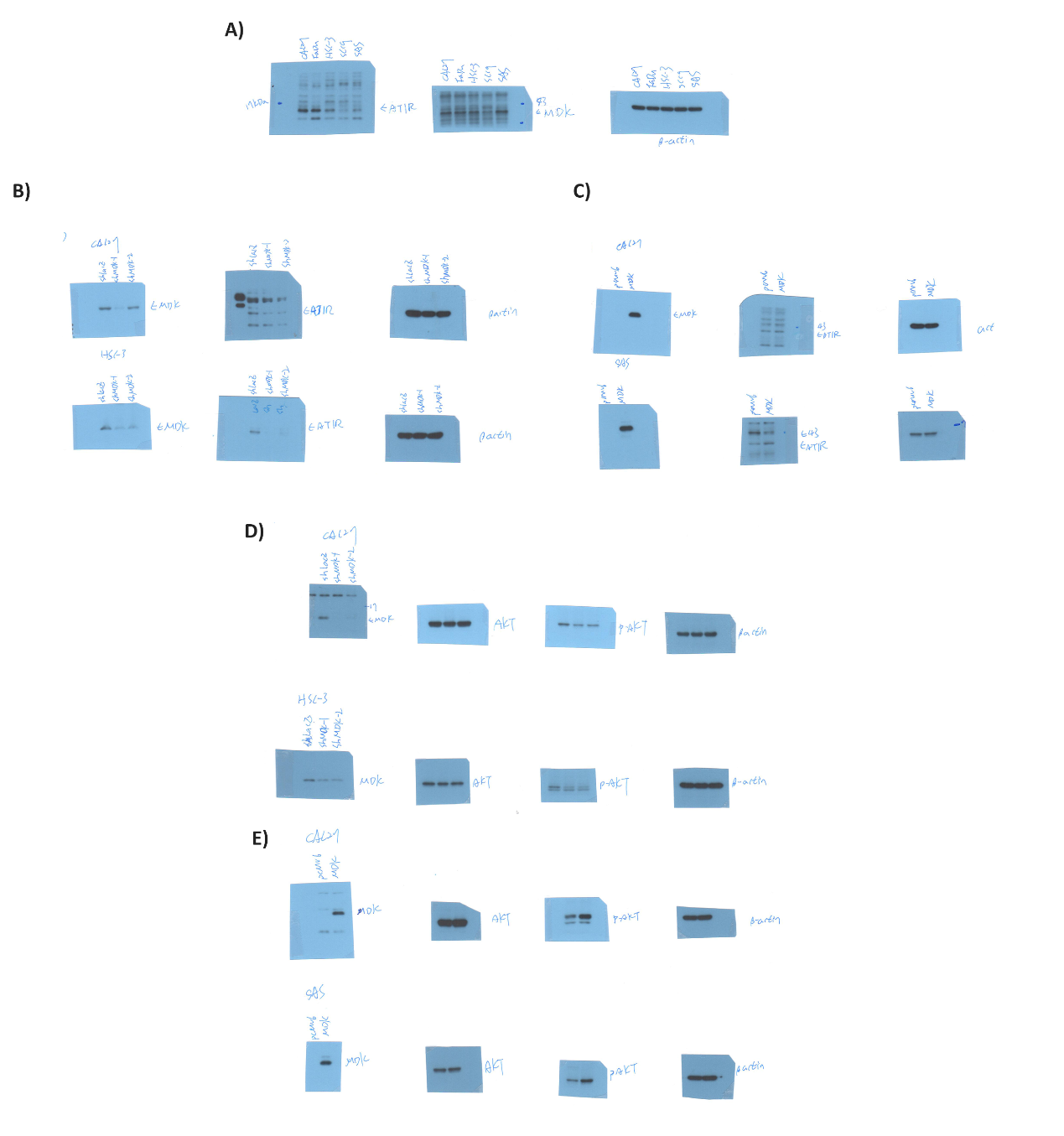


**Figure S2.** The complete image of the Western blot for the Figures 2-5. **A. t**he uncropped blot for fig. 2A. **B.** the uncropped blot for fig. 3A. **C.** the uncropped blot for fig. 4A. **D.** the uncropped blot for fig. 5A. **E.** the uncropped blot for fig. 5B.
